# Development of a key performance indicator for breast cancer in Queensland, Australia

**DOI:** 10.1007/s10549-022-06796-w

**Published:** 2022-11-09

**Authors:** Euan T. Walpole, Philippa H. Youl, Julie Moore, Michelle Morris, Danica Cossio, Pardeep Dhanda, David E. Theile, Shoni Philpot

**Affiliations:** 1 Division of Cancer Services, Princess Alexandra Hospital, Ipswich Road, Brisbane, Woolloongabba, QLD 4102 Australia; 2 The University of Queensland, Brisbane, QLD Australia; 3 Queensland Cancer Control Safety and Quality Partnership, Metro South Hospital and Health Service, Woolloongabba, QLD Australia; 4 Cancer Alliance Queensland, Metro South Hospital and Health Service, Princess Alexandra Hospital, Burke Street, Woolloongabba, Qld 4102 Australia; 5 Sunshine Coast Hospital and Health Service, Sunshine Coast University Hospital, 6 Doherty Street, Birtinya, Qld 4575 Australia; 6 Translational Research Institute, University of Queensland, Brisbane, Qld Australia

**Keywords:** Breast cancer, Treatment, Management, Survival

## Abstract

**Purpose:**

Using population-based data for women diagnosed with stage I-III breast cancer, our aim was to examine the impact of time to treatment completion on survival and to identify factors associated with treatment delay.

**Methods:**

This retrospective study used clinical and treatment data from the Queensland Oncology Repository. Time from diagnosis to completing surgery, chemotherapy and radiation therapy identified a cut-off of 37 weeks as the optimal threshold for completing treatment. Logistic regression was used to identify factors associated with the likelihood of completing treatment > 37 weeks. Overall (OS) and breast cancer-specific survival (BCSS) were examined using Cox proportional hazards models.

**Results:**

Of 8279 women with stage I-III breast cancer, 31.9% completed treatment > 37 weeks. Apart from several clinical factors, being Indigenous (*p* = 0.002), living in a disadvantaged area (*p* = 0.003) and receiving ≥ two treatment modalities within the public sector (*p* < 0.001) were associated with an increased likelihood of completing treatment > 37 weeks. The risk of death from any cause was about 40% higher for women whose treatment went beyond 37 weeks (HR 1.37, 95%CI 1.16–1.61), a similar result was observed for BCSS. Using the surgery + chemotherapy + radiation pathway, a delay of > 6.9 weeks from surgery to starting chemotherapy was significantly associated with poorer survival (*p* = 0.001).

**Conclusions:**

Several sociodemographic and system-related factors were associated with a greater likelihood of treatment completion > 37 weeks. We are proposing a key performance indicator for the management of early breast cancer where a facility should have > 90% of patients with a time from surgery to adjuvant chemotherapy < 6.9 weeks.

## Introduction

Cancer in Queensland, Australia, is a notifiable disease with population wide data available since 1982. While incidence rates have increased, outcomes have improved as measured by five-year survival over the last 20 years [1]. Unfortunately, there is inequity in outcomes with lower socioeconomic groups and Indigenous populations having poorer survival. This gap has not closed despite greater availability of services especially in non-major metropolitan areas. [1, 2]

To better understand the variation in outcomes, significant work linking patient characteristics (demographics, cancer type and stage) with treatment details (surgery, systemic therapy and radiation treatment) and outcome (survival) has resulted in the development of the Queensland Oncology Repository (QOR). This work is performed under the auspices of a government gazetted quality assurance committee, Queensland Cancer Control Safety and Quality Partnership [3]. Legislation allows access to identified cancer patient information—to improve the safety and quality of cancer services through: clinician led service improvement and reform; collection, coordination, analysis, reporting and feedback of cancer data; collaboration on problem solving in the interests of better services for patients and improved outcomes; negotiate development and uptake of strategies to address safety and quality gaps [4].

Breast cancer is the most common cancer in women in Queensland and despite routine screening and improvements in treatment, continues to cause excess deaths. Mulitmodality breast cancer care can negatively influence coordination of treatments and potentially poorer outcomes [5–14]. While most of these studies have examined timing of single therapy modalities (such as time from surgery to beginning adjuvant therapy), Pratt and colleagues recently examined the role of time to treatment completion on survival amongst women receiving surgery, chemotherapy and radiation [15]. They determined a ‘threshold’ of 38 weeks to complete treatment was the most optimal cut-point with clinical meaning. After adjustment for various clinical and sociodemographic variables, results indicated an approximate 20% decrease in overall survival for women whose treatment was completed beyond their 38-week threshold.

In Queensland, cancer care is provided directly by government funded facilities (public) as well as fee for service private facilities (private). Indigenous and low socioeconomic populations tend to be managed within public facilities. Queensland Health as the governing body for public facilities has performance indicators for access to care. [16] The key performance indicators cover access to emergency services, outpatient medical services and surgical waiting times. Cancer is classified as urgent so receives priority at all stages of the diagnostic and treatment pathway. Time measures are from initial referral, thus there is potential for serial delays at points of service access. The Queensland Cancer Quality Index has previously demonstrated these delays in the public system [2].

Using QOR, we have examined the effects of treatment delay on overall and breast cancer-specific survival in a population-based study in Queensland using a similar methodology to that of Pratt et al. [15] The intention was to develop performance indicators for the care of breast cancer patients with the hope to reduce the survival gap for known sociodemographic groups. This would then be incorporated into the quality index and be proposed as a cancer performance indicator to Queensland Health.

## Methodology

This retrospective population-based study using linked data from QOR identified and extracted details for cases of female invasive breast cancer from 2005 to 2015 which allowed a minimum of five years follow up from the diagnostic year. Eligibility criteria included single case of stage I-III breast cancer, with treatment modalities including surgery, and chemotherapy, and radiation therapy. Additionally, all treatment needed to be completed within 18 months of diagnosis. We additionally excluded women with a history of another cancer either prior to or following their breast cancer diagnosis. For women whose time to complete treatment was beyond 12 months, a manual check of pathology and other clinical records was undertaken and women were subsequently excluded where any treatments were for progression or recurrence.

Diagnosis was defined by the date of the histologic confirmation of invasive breast cancer. To calculate the number of days between date of diagnosis and completing treatment, we defined the maximum treatment date using last surgery date (where multiple surgeries had occurred), first chemotherapy start date and radiation therapy end date.

### Calculation of the treatment threshold

The treatment delay threshold of 37 weeks for time to treatment completion was based on results using the empirical cutpoint estimation in Stata. This method estimated the optimal cutpoint with good specificity using the reference variable death at five years post-diagnosis and the classification variable the number of days from diagnosis to end of treatment. We additionally re-ran the analysis using the median and mean values (35 and 36 weeks, respectively) and found no difference in results.

### Variables included

We included age, Indigenous status, type of treatment facility (public or private), and number of comorbidities. Residence at time of diagnosis was categorised into three groups: major city, inner regional and rural (outer regional, remote and very remote) on the basis of the Australian Geographical Classification. [17] Socioeconomic status was assigned according to the Australian Bureau of Statistics Socio-Economic Indexes for Areas (SEIFA). [18] Clinical variables included tumour size, grade and lymph node status. For the time period examined, routine collection of hormone receptor status or HER-2 status was not available.

### Analysis

The statistical significance of bivariate comparisons between women who did and did not complete treatment within 37 weeks and various sociodemographic and clinical factors were estimated using Chi-square or Kruskal–Wallis test. Logistic regression analysis was used to identify factors associated with the likelihood of treatment being completed beyond 37 weeks. Cox Proportional hazard models were used to identify factors independently associated with risk of death from breast cancer and death from all causes at five years post diagnosis. Kaplan–Meier survival curves were constructed to examine five-year overall and breast cancer-specific survival according to time to treatment completion. All analyses were conducted using Stata V17.0 (Stata Corp, College Station, TX).

This study was performed as a quality assurance activity of the Queensland Cancer Control Safety and Quality Partnership.

## Results

Of 31,423 women diagnosed with invasive breast cancer in the years 2005 to 2015, 11,369 (36.2%) received surgery, chemotherapy and radiation therapy and of those 9804 completed their treatment within 18 months of diagnosis. We excluded a further 1525 women who had either stage IV disease, had progression of their breast cancer within 18 months of diagnosis or who had received treatment for another cancer.

Thus, the final cohort included 8279 women of whom, 89.3% (*n* = 7,396) received surgery followed by chemotherapy followed by radiation. Only 7.9% (*n* = 651) had neoadjuvant chemotherapy. The median age was 52. Approximately one-third (31.9%) completed their treatment beyond 37 weeks.

Using a multivariate model (Table 1), we identified several factors significantly associated with an increased likelihood of completing treatment beyond the 37-week threshold. Sociodemographic factors included being Indigenous, living in a middle or disadvantaged area and having two or more comorbidities. There was no association with area of residence. Apart from clinical factors such as higher grade (*p* = 0.003), increasing tumour size (*p* < 0.001) and higher burden of lymph node positivity (*p* < 0.001), we additionally found women who had at least two of their treatment modalities in the public sector were more than twice as likely as those treated in the private sector to complete treatment beyond 37 weeks (OR 2.45, 95%CI 2.22–2.72).

**Table 1:** Sociodemographics and clinical characteristics of 8279 women with stage I-III breast cancer who received surgery, radiation therapy and chemotherapy within 18 months of diagnosis

	Time to complete treatment					Treatment completed > 37 weeks^a^	
	≤ 37 weeks *n* = 5641 (68.1%)	> 37 weeks *n* = 2638 (31.9%)			*p*-value	Adjusted OR (95%CI)	*p*-value
Age at diagnosis					0.03		0.08
< 40	554 (65.7)	289 (34.3)				0.91 (0.77–1.08)	
40–49	1803 (69.9)	775 (30.1)				0.87 (0.78–0.97)	
50–69	2977 (67.9)	1406 (32.1)				Ref	
70 +	307 (64.6)	168 (35.4)				1.04 (0.84–1.28)	
Indigenous status^b^					< 0.001		0.002
Non-Indigenous	5558 (68.6)	2546 (31.4)				Ref	
Indigenous	83 (47.7)	91 (52.3)				1.67 (1.21–2.31)	
Residential location					< 0.001		0.91
Major city	3966 (69.5)	1738 (30.5)				Ref	
Inner regional	1121 (65.9)	580 (34.1)				1.03 (0.90–1.17)	
Rural^c^	554 (63.4)	320 (36.6)				1.03 (0.87–1.21)	
Socioeconomic status					< 0.001		0.003
Affluent	976 (75.9)	310 (24.1)				Ref	
Middle	3623 (67.6)	1737 (32.4)				1.30 (1.12–1.52)	
Disadvantaged	1042 (63.8)	591 (36.2)				1.25 (1.03–1.51)	
Comorbidities					< 0.001		0.03
None	5078 (68.8)	2308 (31.2)				Ref	
One	449 (65.6)	236 (34.4)				1.02 (0.85–1.22)	
Two or more	114 (54.8)	94 (45.2)				1.50 (1.11–2.03)	
Morphology					0.41		0.05
Ductal	4569 (67.9)	2160 (32.1)				Ref	
Lobular	887 (69.7)	386 (30.3)				0.83 (0.72–0.97)	
Other	185 (66.8)	92 (33.2)				0.98 (0.74–1.29)	
Tumour grade					< 0.001		0.003
Grade 1	414 (73.8)	147 (26.2)				Ref	
Grade 2	2262 (68.4)	1045 (31.6)				1.34 (1.08–1.66)	
Grade 3	2830 (67.9)	1341 (32.1)				1.46 (1.18–1.81)	
Not stated/unknown	135 (56.2)	105 (43.8)				1.15 (0.79–1.66)	
Tumour size					< 0.001		< 0.001
≤ 10 mm	551 (73.7)	197 (26.3)				Ref	
11-20 mm	2079 (74.9)	698 (25.1)				0.88 (0.73–1.07)	
21-50 mm	2354 (66.4)	1190 (33.6)				1.06 (0.88–1.28)	
> 50 mm	552 (60.7)	357 (39.3)				1.15 (0.91–1.44)	
Not stated/unknown	105 (34.9)	196 (65.1)				3.68 (2.51–5.40)	
Lymph node status					< 0.001		< 0.001
Negative	2708 (80.3)	663 (19.7)				Ref	
1–3 positive	1870 (63.5)	1074 (36.5)				2.47 (2.19–2.78)	
4–9 positive	625 (56.8)	475 (43.2)				2.99 (2.56–3.50)	
≥ 10 positive	328 (56.3)	255 (43.7)				3.03 (2.48–3.69)	
Not stated/unknown	110 (39.1)	171 (60.9)				2.82 (1.98–4.01)	
Multiple surgeries					< 0.001		< 0.001
No	4421 (69.5)	1944 (30.5)				Ref	
Yes	1220 (63.7)	694 (36.3)				1.42 (1.27–1.70)	
Facility type^d^				< 0.001			< 0.001
Private 2 +	3562 (77.0)		1064 (23.0)			Ref	
Public 2 +	2079 (56.9)		1574 (43.1)			2.45 (2.22–2.72)	

^a^37 weeks identified as optimal cut-point with highest specificity for treatment completion; ^b^Indigenous status unknown for one patient; ^c^Rural includes outer regional, remote and very remote; ^d^facility for 2 or more treatment types

### Survival

**Fig. 1:**
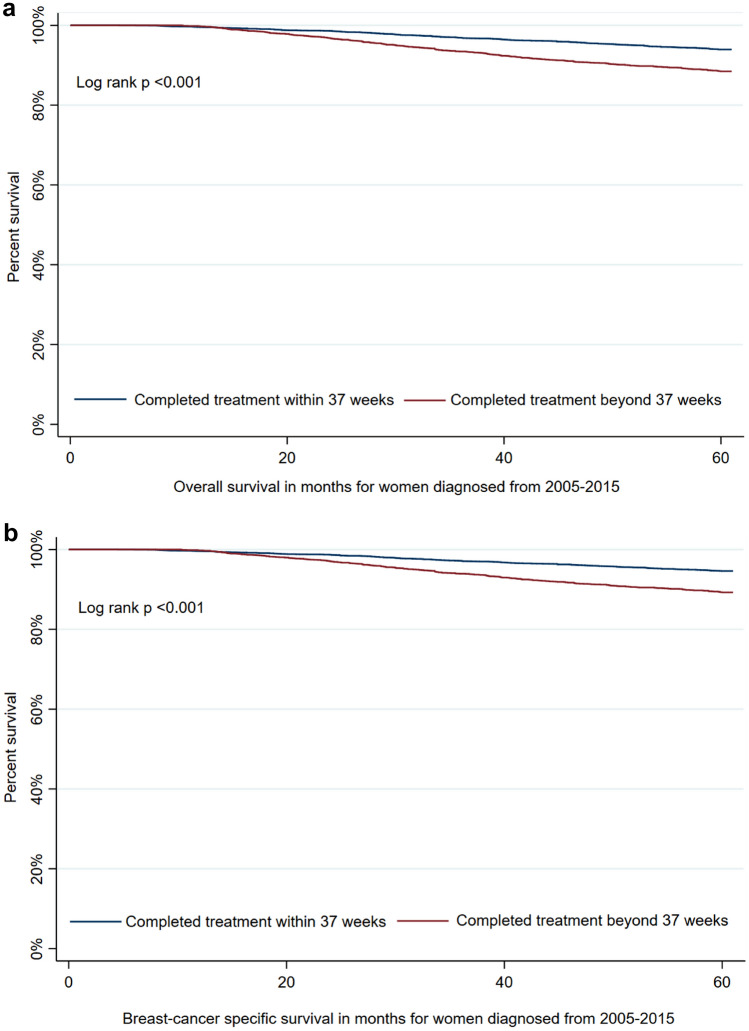
**a** Five-year overall survival by time to treatment completion **b** Five-year breast cancer-specific survival by time to treatment completion

Of the cohort, five-year overall survival was 92.2% (95%CI 91.6–92.8) and breast cancer-specific survival was 92.9% (95%CI 92.3–93.4). Both overall and breast cancer-specific survival were significantly lower for women who completed treatment beyond the 37-week threshold (Figs. 1a, b).

Using a multivariate Cox regression model (Table 2), we examined factors associated with an increased risk of death within five years of diagnosis from any cause as well as death from breast cancer. The risk of death from any cause was higher for women aged 70 + compared to aged 50–69 years and for those living in a socioeconomically middle or disadvantaged compared to an affluent area (*p* < 0.001 and *p* = 0.03, respectively). As expected, several clinical factors were significantly associated with an increased risk of death from any cause or from breast cancer. We found no association with area of residence. Those who received two or more treatment types in a public versus private facility had a 26% increased risk of death from any cause as well as from breast cancer. Women who completed treatment beyond the 37-week threshold were about 37% more likely to die from any cause within five years of diagnosis (OR 1.37, 95%CI 1.16–1.61) and the risk was greater for death from breast cancer (OR 1.43, 95%CI 1.20–1.70). We did however find a reduced risk of death for women who had multiple surgeries—the most common of which was a BCS followed by re-excision (Table 2).

**Table 2:** Multivariate Cox proportional hazards model examining factors associated with higher risk of death within five years of diagnosis from any cause and from breast cancer

	Risk of death from any cause *N* = 645 (7.8%)		Risk of death from breast cancer *N* = 587 (7.1%)	
	HR^a^(95%CI)	*p*-value	HR^a^ (95%CI)	*p*-value
Age at diagnosis		< 0.001		< 0.001
< 40	0.99 (0.77–1.27)		1.01 (0.78–1.32)	
40–49	0.80 (0.66–0.97)		0.84 (0.69–1.03)	
50–69	Ref		Ref	
70 +	1.86 (1.46–2.38)		1.75 (1.34–2.28)	
Indigenous status^b^		0.46		0.51
Non-Indigenous	Ref		Ref	
Indigenous	1.18 (0.76–1.83)		1.17 (0.74–1.85)	
Residential location		0.73		0.67
Major city	Ref		Ref	
Inner regional	0.92 (0.75–1.13)		0.91 (0.73–1.13)	
Rural^c^	0.99 (0.77–1.27)		1.01 (0.78–1.32)	
Socioeconomic status		0.02		0.09
Affluent	Ref		Ref	
Middle	1.41 (1.08–1.83)		1.34 (1.02–1.76)	
Disadvantaged	1.51 (1.11–2.07)		1.39 (1.01–1.92)	
Comorbidities		0.004		0.11
None	Ref		Ref	
One	1.15 (0.89–1.48)		1.06 (0.81–1.39)	
Two or more	1.75 (1.25–2.44)		1.49 (1.03–2.16)	
Morphology		0.26		0.38
Ductal	Ref		Ref	
Lobular	1.05 (0.83–1.33)		1.10 (0.86–1.41)	
Other	1.38 (0.93–2.03)		1.30 (0.85–1.97)	
Tumour grade		< 0.001		< 0.001
Grade 1/2	Ref		Ref	
Grade 3	2.53 (2.09–3.05)		2.91 (2.38–3.57)	
Not stated/unknown	2.26 (1.50–3.39)		2.43 (1.58–3.72)	
Tumour size		< 0.001		< 0.001
≤ 10 mm	Ref		Ref	
11-20 mm	1.19 (0.79–1.81)		1.14 (0.74–1.76)	
21-50 mm	1.65 (1.11–2.46)		1.55 (1.03–2.35)	
> 50 mm	2.46 (1.61–3.77)		2.36 (1.52–3.66)	
Not stated/unknown	1.54 (0.85–2.82)		1.58 (0.85–2.93)	
Lymph node status		< 0.001		< 0.001
Negative	Ref		Ref	
1–3 positive	1.64 (1.30–2.08)		1.74 (1.35–2.24)	
4–9 positive	3.19 (2.49–4.08)		3.36 (2.59–4.38)	
≥ 10 positive	4.69 (3.60–6.10)		5.13 (3.89–6.78)	
Not stated/unknown	3.47 (2.12–5.67)		3.57 (2.13–5.98)	
Multiple surgeries		< 0.001		< 0.001
No	Ref		Ref	
Yes	0.69 (0.56–0.86)		0.65 (0.52–0.82)	
Facility type^d^		0.005		0.009
Private 2 +	Ref		Ref	
Public 2 +	1.26 (1.07–1.49)		1.26 (1.06–1.49)	
Treatment completed > 37 weeks		< 0.001		< 0.001
No	Ref		Ref	
Yes	1.37 (1.16–1.61)		1.43 (1.20–1.70)	

^a^Hazard ratio; ^b^Indigenous status unknown for one patient; ^c^Rural includes outer regional, remote and very remote; ^d^facility for 2 or more treatment types

To help identify whether there was a particular part of the treatment pathway that contributed most to poorer outcomes, we further examined the 7302 women who had received surgery, followed by chemotherapy followed by radiation therapy. Using the same methodology as for the main analysis, we determined the optimal cut-points (or thresholds) to start each treatment modality. From diagnosis to surgery the optimal threshold was 2.3 weeks (16 days), for last surgery to starting chemotherapy it was 6.9 weeks (48 days) and from completing chemotherapy to completing radiation it was 12.5 weeks (88 days).

The thresholds for each of the treatment modalities were included as covariates in a Cox model to examine factors associated with risk of death from breast cancer (Table 3). While several clinical prognostic factors were significantly associated with an increased risk of death from breast cancer, we were unable to examine the impact of tumour hormonal status or HER-2 positivity due to lack of data. We additionally found an approximate 40% increased risk (Hazard ratio = 1.42, 95%CI 1.18–2.10) of death from breast cancer for women whose time from last surgery to starting CT was beyond the threshold of 6.9 weeks (approximately 48 days). We did not observe any significant association with time from diagnosis to first surgery nor for the time from completing chemotherapy to completing radiation therapy. Area-level disadvantage and treatment in the public system were no longer associated with an increased risk of death (Table 3).

**Table 3:** Multivariate Cox proportional hazards model examining factors associated with higher risk of death within five years of diagnosis from breast cancer for 7396 women who received surgery → chemotherapy → radiation therapy

	Risk of death from breast cancer *N* = 424 (5.7%)	
	HR^a^(95%CI)	*p*-value
Age at diagnosis		0.004
< 40	1.06 (0.78–1.43)	
40–49	0.84 (0.67–1.07)	
50–69	Ref	
70 +	1.64 (1.18–2.29)	
Indigenous status^b^		0.48
Non-Indigenous	Ref	
Indigenous	1.22 (0.70–2.11)	
Residential location		0.87
Major city	Ref	
Inner regional	0.94 (0.73–1.21)	
Rural^c^	0.95 (0.69–1.32)	
Socioeconomic status		0.23
Affluent	Ref	
Middle	1.31 (0.96–1.81)	
Disadvantaged	1.32 (0.91–1.93)	
Comorbidities		0.06
None	Ref	
One	1.04 (0.75–1.44)	
Two or more	1.71 (1.10–2.65)	
Tumour grade		< 0.001
Grade 1/2	Ref	
Grade 3	3.49 (2.75–4.43)	
Not stated/unknown	2.66 (1.12–6.31)	
Tumour size		< 0.001
≤ 10 mm	Ref	
11-20 mm	1.79 (0.95–3.35)	
21-50 mm	2.48 (1.35–4.56)	
> 50 mm	3.58 (1.89–6.76)	
Not stated/unknown	2.49 (0.63–9.77)	
Lymph node status		< 0.001
Negative	Ref	
1–3 positive	1.71 (1.29–2.62)	
4–9 positive	3.53 (2.63–4.74)	
≥ 10 positive	5.40 (3.96–7.37)	
Not stated/unknown	0.83 (0.64–2.81)	
Multiple surgeries		0.02
No	Ref	
Yes	0.73 (0.57–0.94)	
Facility type^d^		0.13
Private 2 +	Ref	
Public 2 +	1.20 (0.95–1.51)	
Diagnosis to first surgery > 2.3 weeks	1.07 (0.88–1.31)	0.95
Last surgery to start CT > 6.9 weeks	1.42 (1.18–2.10)	0.001
End CT to end RT > 12.5 weeks	1.24 (0.98–1.58)	0.10

^a^Hazard ratio; ^b^Indigenous status unknown for one patient; ^c^Rural includes outer regional, remote and very remote; ^d^facility for 2 or more treatment types

**Fig. 2:**
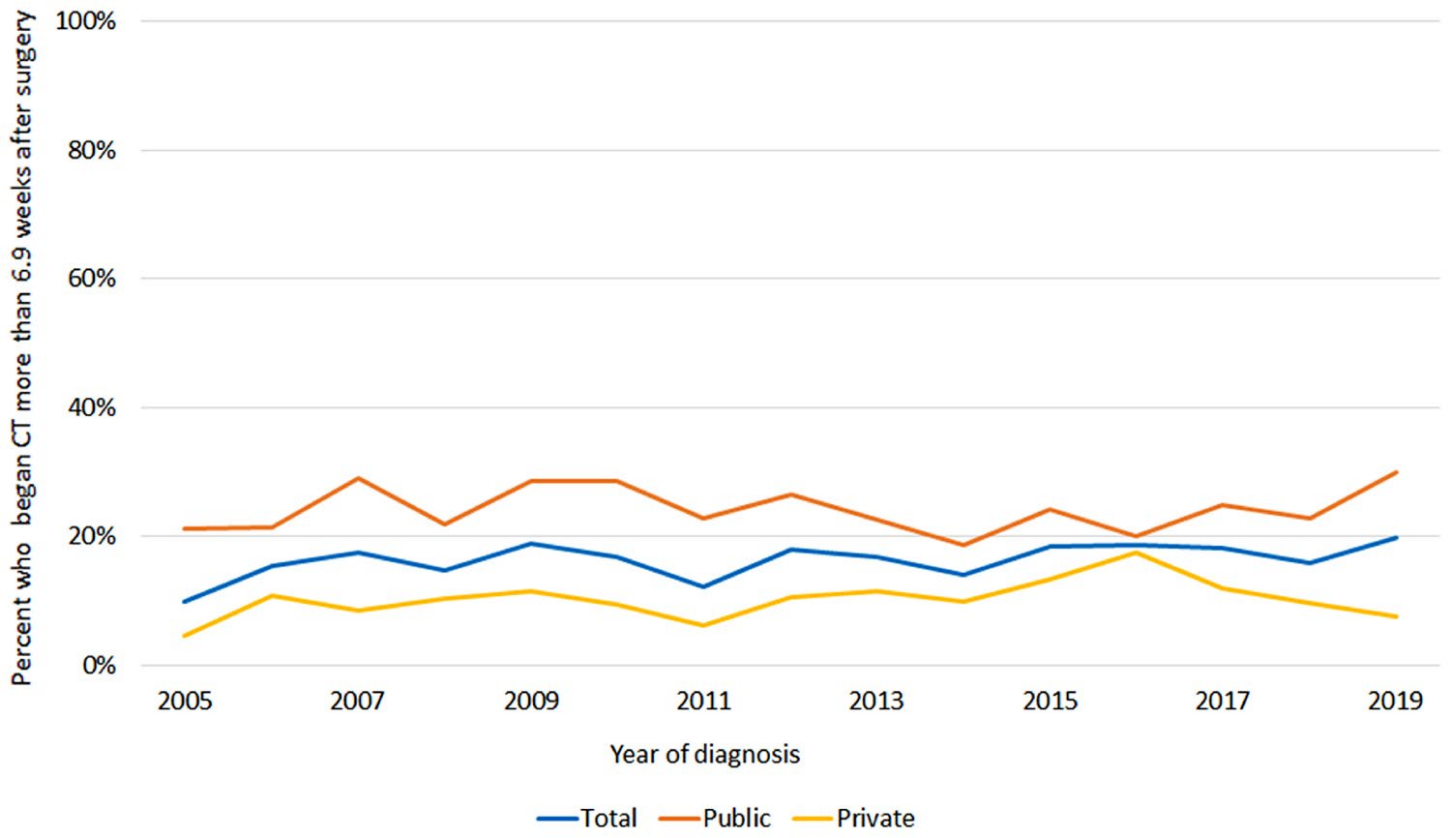
Proportion of women experiencing delay to start chemotherapy following surgery

As an exploratory option, the proportion of women completing treatment beyond 37 weeks was extended into more recent cohorts where long term follow up was not available. We found that the proportion of women with chemotherapy delay is an increasing issue for the public sector. (Fig. 2).

**Fig. 3:**
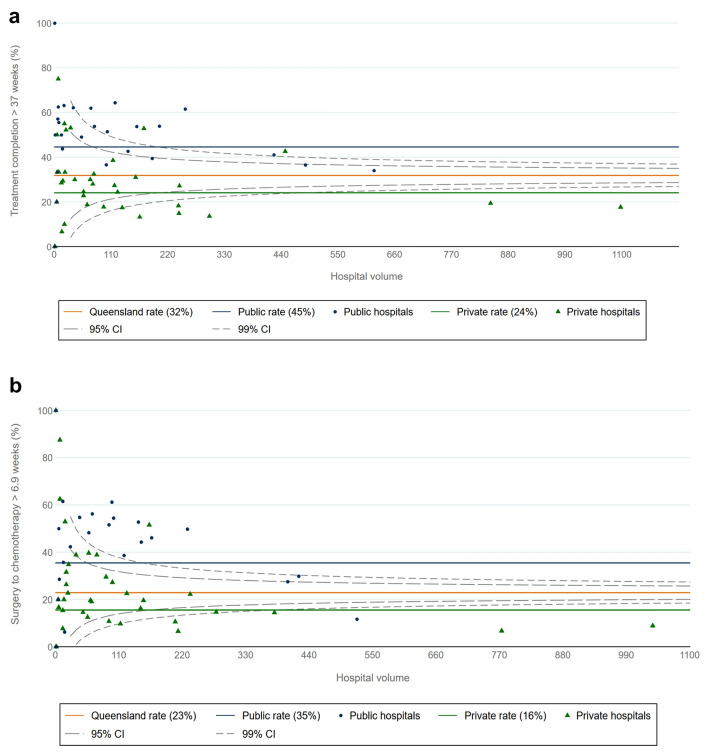
**a** Funnel plot of surgery → chemotherapy → radiation delay > 37 weeks by treatment site and site volume. **b** Funnel plot of surgery → chemotherapy delay by treatment site and site volume

Whilst overall delay in treatment is linked to impaired survival, it can only be examined retrospectively. Delay to each aspect of care is potentially able to be monitored and modified as a performance indicator. We performed funnel plots comparing the proportion of cases with treatment completion > 37 weeks by treatment facility and volume as well as a similar plot of proportion of cases with time from surgery to chemotherapy. (Fig. 3a, b) The treatment sites out of range are similar in both plots suggesting that chemotherapy delay is an early warning for prolonged treatment completion and poorer survival given the results of the multivariate analysis.

## Discussion

In this population-based study of over 8000 women with stage I-III breast cancer we found nearly one-third experienced a delay in completing treatment. Our analysis found women who did not complete treatment within the 37-week threshold had significantly poorer five-year overall and breast cancer-specific survival. Our results are comparable to that of Pratt and colleagues who found poorer survival for women who completed treatment beyond their 38-week threshold [15].

While it is widely known that delays in commencing adjuvant treatment for breast cancer result in poorer survival, very few studies have examined the impact of delay where multimodality treatment is employed, particularly at a population level. Likely this is due to the lack of linked data that includes sociodemographic, clinical as well as start and completion dates for each treatment modality. In Queensland, our web based system, Queensland Oncology Online (QOOL©), links patient demographic, diagnosis, stage and treatment data. This provides an automated method for measuring delay that is then visualised for each site via business intelligence software as a performance indicator. The QOOL system can be applied to historical and new cases to identify and manage treatment delay.

We found some sociodemographic groups were more likely to experience a delay in completing treatment, including Indigenous women and those living in disadvantaged areas. These findings are similar to others showing a higher likelihood of treatment delays in Indigenous ethnic groups and for the disadvantaged. [8, 19–21] While the reasons for treatment delays are likely to be multifactorial including system-related factors, lower levels of education and health literacy found more commonly amongst Indigenous and disadvantaged women may be one factor contributing to treatment delays. [8] We were however unable to measure these factors in this study.

Receiving two or more treatment modalities in the public, compared to the private system, was associated with a more than twofold higher risk of treatment completed beyond the 37-week threshold. While it could be argued that this finding is likely due to variations in the casemix of patients, our analysis was fully adjusted for several clinical factors. We did not find an independent association between area of residence and the likelihood of delay in treatment completion, however rural location has previously been found to increase the likelihood of delays in treatment [21]. In this study about two-thirds of rural women attended a public facility for their treatment compared to about 40% of urban women. The complexity of coordinating multimodality therapy within the public system may be one reason for this finding, in addition to a continued growing need for oncology services. Care coordination is routinely available to manage individual care pathways but this data would suggest it has not delivered the expected outcomes.

Whilst delays may adversely cause women who are waiting for treatments increasing anxiety, do these delays result in poorer outcomes? In this study we found a significant association between longer time to treatment completion and reduced overall and breast cancer-specific survival, after adjustment for various tumour characteristics. Our results are similar to Pratt and colleagues who found an approximate 20% increased risk of death within five years of diagnosis for women whose treatment went beyond their threshold of 38 weeks [15]. While there are guidelines in Australia relating to timing of surgery (within four weeks of initial consultation), adjuvant chemotherapy (4–6 weeks from surgery date) and radiation therapy (within 8 weeks of surgery or within 3–4 weeks of completion of adjuvant chemotherapy), [22] no specific recommendation exists regarding the optimal time to complete all three treatment modalities. Identifying the time points relating to the various treatment modalities and their influence on survival is important if we are going to reduce the inequities in survival.

In our study we found the critical point which appeared to have the most influence on poorer survival is the time from surgery to beginning chemotherapy. In our analysis we identified the threshold from surgery to beginning chemotherapy was 6.9 weeks (48 days). Several studies have identified delays in adjuvant therapy and poorer survival with most finding associations with a delay of > 90 days from surgery to beginning chemotherapy increased the risk of death between 20 and 30%. [6, 7, 23] He and colleagues [10] conducted a meta-analysis involving nearly 187,000 patients and found factors associated with delay to begin adjuvant chemotherapy included living in a rural area and receiving mastectomy rather than breast conserving surgery. Meyer et al. also found inclusion in a therapeutic trial was associated with delays in initiating adjuvant chemotherapy, [12] and a further study identified older age and non-English language were associated with prolonged interval from surgery to starting chemotherapy. [24] We performed some additional analysis to help identify factors associated with surgery to beginning chemotherapy beyond our threshold of 6.9 weeks. Identified factors included living in a disadvantaged area and having a comorbidity. While delay initiating chemotherapy was more common for rural women, this was not the case when we adjusted for disease stage. The strongest predictor of delay in beginning adjuvant chemotherapy was observed for women who received treatment in a public rather than private facility.

Whilst treatment advances are important in cancer outcomes in the future, it is providing optimal evidence-based care that may reduce the present differences seen by socioeconomic and ethnic variations. Despite this knowledge being available for years, this disparity remains and interventions appear to have failed.

Cancer Alliance Queensland will continue to monitor treatment completion > 37 weeks and time from surgery to chemotherapy > 6.9 weeks on a population wide basis. The funnel plots will become part of the reporting on breast cancer treatment in Queensland. In-depth reports are provided to clinicians, administrators, and individual hospital quality assurance committees for review and to stimulate service improvement. We are proposing that the public health system manager accepts a key performance indicator for the management of early breast cancer where a facility should have > 90% of patients with a time from surgery to adjuvant chemotherapy < 6.9 weeks. We intend to monitor and report this measure which will demonstrate whether there is a decrease in the delay to chemotherapy as well as a subsequent reduction in the proportion of women who completed treatment > 37 weeks.

## Limitations

Whilst a strength of this study is its population-based nature and inclusion of linked data from several sources, some limitations need to be considered. Our study included a comprehensive suite of clinical factors however, we did not have complete data on hormone status or HER-2 status of the tumour. However, Pratt et al. found the poorer survival for those who completed treatment beyond their 38-week threshold did not vary according to hormone status [15]. Further, we did not have access to data on hormonal therapy nor did we have complete data on chemotherapy regimens or detailed information on radiation fractions. We are however in the process of linking more detailed information on the treatments received. The role of patient preference for timing of therapies was also not able to be assessed.

Neoadjuvant treatment of breast cancer was uncommon at the start of the study period except for locally advanced tumours. In the future, we will need to re-examine the treatment pathway impact including the change in order of treatment in a reanalysis linking with complete hormonal and HER-2 status data. Pathologic response to neoadjuvant therapy is likely to add a new important variable for analysis.

## Conclusions

This study found time to treatment completion beyond 37 weeks was associated with poorer overall and breast cancer-specific survival. Apart from clinical factors, we identified several sociodemographic and system-related factors were associated with a greater likelihood of delay in completing treatment for breast cancer. Delay from surgery to beginning adjuvant chemotherapy appears to be the point that most strongly influences survival. Whilst the coordination of multimodality therapy can be complex, identifying and addressing the factors that influence delay is critical to reduce to survival inequalities.

## Data Availability

The datasets generated during and/or analysed during the current study are not publicly available due to confidentiality but are available from the corresponding author on reasonable request.
